# Quantitative titanium imaging in fish tissues exposed to titanium dioxide nanoparticles by laser ablation-inductively coupled plasma-mass spectrometry

**DOI:** 10.1007/s00604-023-05895-9

**Published:** 2023-07-18

**Authors:** Cristian Suárez-Oubiña, Annarosa Mangone, Lorena C. Giannossa, Laura Nuñez-González, Paloma Herbello-Hermelo, Pilar Bermejo-Barrera, Antonio Moreda-Piñeiro

**Affiliations:** 1 Trace Element, Spectroscopy and Speciation Group (GETEE), Institute of Materials (iMATUS), Department of Analytical Chemistry, Nutrition and Bromatology, Faculty of Chemistry, Universidade de Santiago de Compostela, Avenida das Ciencias, s/n, 15782 Santiago de Compostela, Spain; 2 Dipartimento di Chimica, Università Aldo Moro, via Orabona 4, 70126 Bari, Italy; 3 Grupo de Genética y Biología del Desarrollo de las Enfermedades Renales, Laboratorio de Nefrología (n.11), Instituto de Investigación Sanitaria (IDIS), Complexo Hospitalario de Santiago de Compostela (CHUS), 15706 Santiago de Compostela, Spain

**Keywords:** Quantitative imaging, Nanoparticles, Gelatine standards, Fish tissues, Laser ablation

## Abstract

Imaging studies by laser ablation–inductively coupled plasma mass spectrometry have been successfully developed to obtain qualitative and quantitative information on the presence/distribution of titanium (ionic titanium and/or titanium dioxide nanoparticles) in sea bream tissues (kidney, liver, and muscle) after exposure assays with 45-nm citrate-coated titanium dioxide nanoparticles. Laboratory-produced gelatine standards containing ionic titanium were used as a calibration strategy for obtaining laser ablation–based images using quantitative (titanium concentrations) data. The best calibration strategy consisted of using gelatine-based titanium standards (from 0.1 to 2.0 μg g^−1^) by placing 5.0-μL drops of the liquid gelatine standards onto microscope glass sample holders. After air drying at room temperature good homogeneity of the placed drops was obtained, which led to good repeatability of measurements (calibration slope of 4.21 × 10^4^ ± 0.39 × 10^4^, *n* = 3) and good linearity (coefficient of determination higher than 0.990). Under the optimised conditions, a limit of detection of 0.087 μg g^−1^ titanium was assessed. This strategy allowed to locate prominent areas of titanium in the tissues as well as to quantify the bioaccumulated titanium and a better understanding of titanium dioxide nanoparticle spatial distribution in sea bream tissues.

**Graphical abstract:**

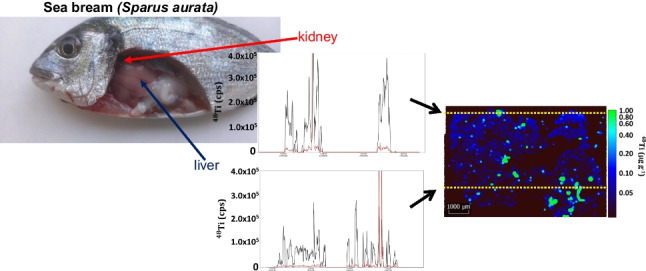

**Supplementary Information:**

The online version contains supplementary material available at 10.1007/s00604-023-05895-9.

## Introduction

Downscaling materials to the nanometre range significantly alters their properties and give rise to interesting new features that allow new and outstanding applications in several industrial sectors. Titanium dioxide nanoparticles (TiO_2_ NPs) are one of the most used engineered nanoparticles (ENPs), and they can be mainly found in personal care products and foodstuff [1, 2]. Therefore, there is great concern about TiO_2_ NP potential toxicity and impact on the environment (aquatic fate and ecotoxicity) and on humans [3, 4].

In addition to the well-established analytical techniques for assessing NPs in environmental (biota) matrices [3], analytical techniques that allow the assessment of elemental distributions in biological tissues may contribute to a better understanding of the presence and degree of affectation of NPs in biota. Imaging for elemental distribution in biological tissues has been reported for trace elements in human tumour samples by laser-induced breakdown spectroscopy (LIBS) and laser ablation–inductively coupled plasma–mass spectrometry (LA-ICP-MS) [5], and for nanosilver-coated bone implants by X-ray fluorescence (XRF) [6]. As an advantage, LA-ICP-MS provides high-quality features, such as a wide dynamic range, multi-element detection capabilities, and high spatial resolution; moreover, sample preparation is less laborious and time consuming than other imaging techniques [5, 7–11]. Imaging studies based on LA-ICP-MS could be a breakthrough aiming the visualisation of metal distribution in biological tissues by using not only qualitative information (relative elemental intensities) but also quantitative data (elemental concentrations)**.**

Applications of LA-ICP-MS in biological tissues have been mainly focused on bioimaging elements in cells [12, 13], tumour tissues [14, 15], brain sections [16], and tissues from several organs [17–19]. Developments for NP imaging by LA-ICP-MS are however scarce [20, 21], mainly when the studies are focused on biodistribution investigations [22–24]. By other side, the heterogeneity of biological samples and the absence of appropriate reference materials for calibration give rise to several difficulties derived from LA-ICP-MS analysis such as the signal drift over time as well as problems of transport and ionisation at the ICP source [25]. Also, the absence of adequate reference materials or standard matrix-matched materials hinders LA-ICP-MS analysis to obtain quantitative imaging studies. Regarding this barrier, some strategies have been proposed, although there are still disagreements to establish a universal calibration or protocol for an efficient quantification strategy [7, 26]. Some proposals for quantitative analysis by LA-ICP-MS include isotope dilution analysis [27] and internal standardisation [28]. As an alternative, lab-produced standards based on gel substances are gradually spreading, especially standards made of porcine gelatine [25, 29], which results adequate for imaging of biological tissues.

The current research has been focused on novel quantitative imaging studies by LA-ICP-MS for assessing the biodistribution of titanium in sea bream tissues (kidney, liver, and muscle) after bioaccumulation assays at TiO_2_ NP concentrations higher than those found in clean seawater (higher than TiO_2_ NP background concentrations). This fish species was selected since it is one of the main farmed fish species produced in EU (93,131 tonnes in 2020) [30] and the aquaculture sector is concerned about the possible impact of the presence of NPs in culture waters. Sea bream tissues were subjected to paraffin polymer embedding and thin slice cutting by microtome as a sample pre-treatment. A calibration technique was established by using lab-produced porcine gelatine standards, which allowed quantitative data-based images. Since the multi-element capabilities of ICP-MS, images obtained from several isotopes of titanium (^48^Ti and ^46^Ti) and control isotopes (mainly ^26^Mg for verifying soft tissue ablation) were simultaneously obtained.

## Experimental

### Instrumentation

A NexION 2000 inductively coupled plasma mass spectrometry (Perkin Elmer, Waltham, MA, USA) equipped with an ESI NWR 213 laser ablation system (ESI New Wave Research Co., Cambridge, UK) was used. The instrument is equipped with triple nickel cone interface and a quartz torch with a quartz injector tube (2.5 mm i.d.). Data acquisition and management were performed with the Syngistix™ Application 2.5 version software (PerkinElmer), which allows data visualisation as it is being acquired in real time. Laser ablation equipment control and management were also performed with ActiveView2 4.1.2 version and data reduction software Iolite4 from Elemental Scientific (NE, USA). An USC-TH ultrasound water bath (45 Hz, 80 W) from VWR International Eurolab S.L (Barcelona, Spain) was used for dispersing NPs before analysis. UV lamp from Vilber Lourmat™ (Marne-la-Vallee, France) was used to visualise fixed fish tissues, whereas ARE heating and magnetic stirrer from Velp Scientifica (MB, Italy) was used for preparing gelatine standards. A pH-metre model Instruments XS (Carpi Mo, Italy) was used.

### Material and reagents

All solutions were prepared with ultrapure water (18.2 MΩ cm of resistivity) obtained from a Milli-Q® IQ 7003 purification device system (Millipore, Bedford, MA, USA). Argon (99.998%) and helium (99.999%) were from Nippon Gases (Madrid, Spain). Mono-elemental 1000 mg L^−1^ standard of titanium [(NH_4_)_2_TiF_6_] was from PerkinElmer.

Citrate-coated TiO_2_ NPs with a primary size of 45 nm (45 nm TiO_2_ NPs) stock dispersions were prepared from pristine 45 nm TiO_2_ NPs [99.5% purity, mixture of rutile and anatase; nanoparticle sizes of < 100 nm (BET) and < 50 nm (XRD)] from Sigma-Aldrich (Osterode, Germany). The 45-nm TiO_2_ NPs were stabilised with trisodium citrate dehydrate aqueous solution reaching a weight ratio of 1:1.5 TiO_2_:citrate. The mixture was dispersed for 30 min using an ultrasonic probe (Branson Disintegrator Ultrasonic Mod. 450; with 30 s pulse on/5 s pulse off, and 50% amplitude). The final concentration of citrate-coated TiO_2_ NPs was 13.3–15.5 g L^−1^ depending on the batch. Figure S1 (Electronic Supplementary Information, ESI) shows a typical TEM image from the prepared material.

Other reagents as sodium hydroxide, sodium hydrogen carbonate, hyperpure nitric acid 69% (w/v), and absolute ethanol were from Panreac (Barcelona, Spain). Porcine-skin gelatine, type A, bloom strength 300, and formaldehyde solution (36.5–38% in water) were from Sigma-Aldrich (Osterode, Germany). Glass sample holders were from Labbox (Barcelona, Spain). To avoid metal contamination, all glassware and plastic ware were washed with ultrapure water and kept in 10% (v/v) nitric acid for 48 h, and then rinsed several times with ultrapure water before use.

### Aquaculture products: exposure experiments and sample preparation

Sea bream (*Sparus aurata*) exposure assays with 45-nm TiO_2_ NPs were carried out by personnel qualified in animal experimentation in authorised facilities of Centro Tecnológico de Acuicultura, CETGA (Ribeira, A Coruña, Spain). All experimental procedures were carried out in accordance with European Union and Spanish Regulations (Council Directive 2010/63/EU (European Union 2010) and R.D. 53/2013 (BOE 2013), respectively), for the protection of animals used for experimental purposes.

Commercial fish feed pellets (Biomar Iberia, S.A.) were used for feeding sea breams along the bioaccumulation assay. Before TiO_2_ NP incorporation to food, a premixture was prepared by combining micronised calcium carbonated and TiO_2_ NPs at an equivalent of 5% of the weight of pellets. The premixture was then added to commercial fish feed pellets for pellet coating until achieving 0.25, 0.75, and 1.5 mg kg^−1^ of fish per day. Feed used in the control group (unexposed specimens) was also coated with micronised calcium carbonated but without NPs.

Sea bream specimens (one hundred and twenty individuals in each tank, average initial weight of 7.7 g, and an age of 147 days after hatching) were daily fed with feed pellet containing 45-nm TiO_2_ NPs at three 45-nm TiO_2_ NP concentration levels (0.25, 0.75, and 1.5 mg kg^−1^, concentrations expressed as mg of TiO_2_ NPs per fish feed mass in kg) for 90 days (samplings were performed each 15 days establishing six different exposure times: 0, 15, 30, 45, 60, 75, and 90 days). Sea breams followed the standard aquaculture procedure for their growth under controlled conditions and were fasted for 1 day before being killed with an overdose of anaesthetic. Different sections (muscle-skin, liver, and kidney) from three fishes under each exposure conditions (TiO_2_ NP concentration and exposure time) were obtained. The samples were frozen and preserved at – 20 °C until analysis.

### Fish tissue preparation

Kidney, liver, and muscle tissues were from sea bream specimens exposed to 45-nm TiO_2_ NPs at different times (up to 90 days) and from unexposed sea bream (control samples). Sample preparation consisted of immersing the fish tissues (kidney, liver, and muscle) in 4% paraformaldehyde at pH 7.0 overnight and 4 °C to harden the tissue (fixation process). The tissues were then subjected to a three-stage washing step (dehydration process) with distilled water, 50% ethanol, and 70% ethanol (the tissues were soaked in each solvent for 15 min), and then, the tissues were kept in 70% ethanol indefinitely before being embedded in blocks of paraffin polymer. The paraffin blocks were cut into 5.0-μm thin slides using a standard microtome, obtaining at least 5 replicates per sample to have enough samples to test and optimise the developed methodology as well as for the final application for quantitative imaging. Finally, the 5.0-μm slices were individually placed on microscope glass sample holders. Additionally, paraffin blocks with no-embedded sample were also prepared and slices were also cut as used as blanks. All paraffin slides (Fig. S2, ESI) were covered with glass sample holders and kept at room temperature before analysis.

### Gelatine standard preparation

Lab-produced gelatine standards were prepared by dissolving gelatine porcine skin (500 mg) in 5.0 mL of ultrapure water (gelatine concentration at 10% (m/v)). Porcine gelatine mixtures were then heated at 70 °C (use of a magnetic heater) until complete liquefaction of the mixture, followed by homogenisation under continuous and soft stirring for 10 min. Liquid paraffin–based titanium standards from 0.1 to 2.0 μg g^−1^ (drops of 5.0 μL) were carefully pipetted (avoiding bubble formation) and displayed onto microscope glass sample holders. Standards were covered and left to dry at room temperature. The titanium-based gelatine standards were analysed in triplicated by LA-ICP-MS.

### LA-ICP-MS measurements

The assessment of Ti spatial distribution concentrations in fish tissues was performed by LA-ICP-MS under optimised operating conditions summarised in Table S1 (ESI). The instrument was daily tuned following the standard procedure based on ablating a NIST SRM 612 standard (50 μm diameter spot, repetition rate of 20 Hz, scan speed of 20 μm s^−1^, and laser energy of 10.0 J cm^−2^ in line-scanning mode). Nebulisation gas flow rate and ICP-MS parameters were also daily tuned by monitoring and verifying intensities as follows: ^9^Be (≥ 15,000 counts), ^24^Mg (≥ 270,000 counts), ^115^In (≥ 400,000 counts), ^208^Pb (≥ 235,000 counts), ^238^U (≥ 400,000 counts), ^232^Th^16^O/^232^Th (< 0.02), ^232^Th/^238^U (> 0.07), and background (< 3.0). In addition to Ti isotopes (^46^Ti, ^47^Ti, ^48^Ti, and ^49^Ti), other isotopes such as ^25^Mg, ^26^Mg, ^31^P, and ^42^Ca were monitored to ensure fish tissue ablation, and ^27^Al to ensure non-ablation of the glass slide.

### Data treatment

Spectra, data analysis, and quantitative images were obtained using the data reduction software Iolite4. NetCDF files from ICP-MS and laser log files from laser ablation equipment were exported from the instrumentation software and attached to Iolite4, aiming optimum results after performing some steps: baseline subtraction, quantification using gelatine standards, assessment of the obtained spectra channels (elements selected in the developed methodology), and image construction selecting CellSpace as a map type. Data tables were also exported as Excel files.

## Results and discussion

### Methodology development

#### Preliminary studies

Based on the available literature, low laser energy conditions and a relatively high scanning rates are recommended for LA-ICP-MS when analysing thin slides of biological tissues polymer embedded [14, 18, 21, 31, 32]. The use of soft ablation conditions by scan line as an ablation work mode is specially needed when analytes such as NPs must be imaged since longer periods of time laser incidence and too high laser energy would lead to NP ionisation resulting in a continuous signal (smoother discrete signals) [21] instead of well-defined and recognised peaks in the continuous LA-ICP-MS record. However, there are many discrepancies in the published literature regarding the influence of other parameters (spot size, frequency, dwell time, and helium flow) on metal imaging by LA-ICP-MS and the significant differences can be attributed to the different capabilities of the laser instrumentation.

Several titanium isotopes (^46^Ti, ^47^Ti, ^48^Ti, and ^49^Ti) were monitored but only ^48^Ti (most abundant isotope) was used for quantification (other minor isotopes were used as qualifier isotopes to verify the presence of titanium). In addition, a preliminary selection of other elements, as control isotopes, was performed for establishing the borders between paraffin-embedded tissue and paraffin-free tissue, and also to control the avoidance of glass slide ablation. Minor isotopes such as ^25^Mg and ^26^Mg, ^31^P, and ^42^Ca (Table S1, ESI) were selected as controls to ensure the ablation of the embedded fish tissue, whereas ^27^Al was used to detect the ablation of the glass slide. The potential ablation of the glass holder can be also observed in the exported images (ActiveView™ software) such as those shown in Fig. S3 (ESI) where a second black line appears in the scan line when ablating the glass slide in addition to the fish tissue polymer-embedded and paraffin polymer. Exported images from ActiveView™ software help also to visualise the borders between paraffin-embedded tissue/paraffin-free tissue and the glass holder (Fig. S4C, ESI) and to show that the embedded biological tissue is not perfectly homogeneous (Fig. S4A, B, ESI).

#### Laser ablation operation parameters

**Fig. 1:**
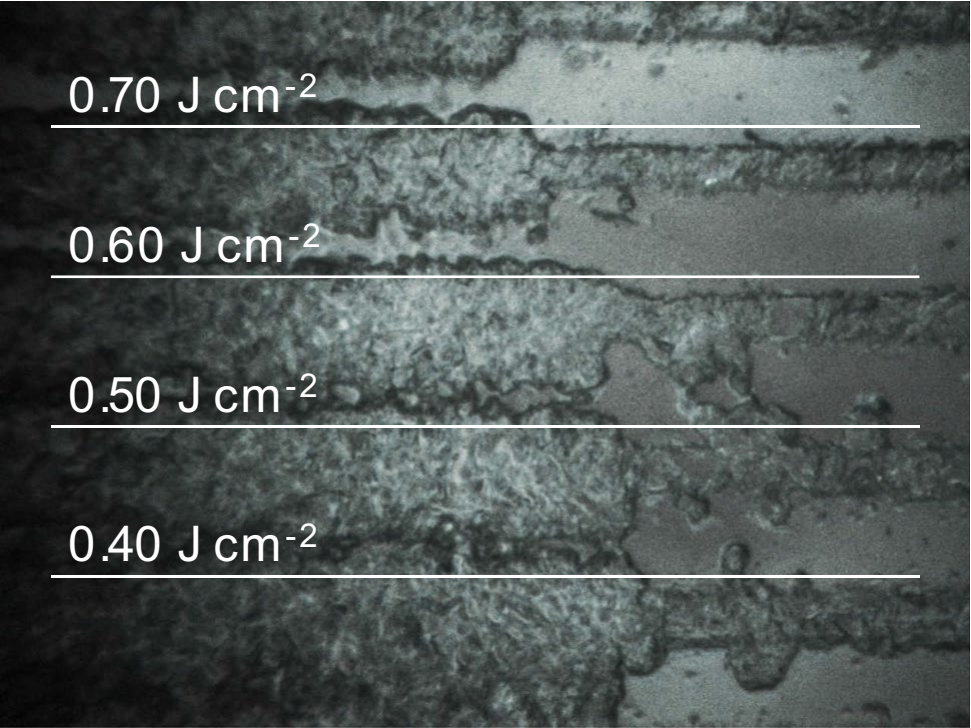
Exported image from ActiveView™ software illustrating the effect of the laser fluency on the ablation of the embedded fish tissue and embedded fish tissue plus paraffin

Experiments at high fluencies or laser energies (> 0.50 J cm^−2^) led to a partial ablation of the glass holder since the increase of the registered ^30^Al intensities (ActiveView™ exported images in Fig. 1), and the laser energy was therefore fixed at 0.40 J cm^−2^, which is a rather low energy but enough to ablate the sample without a strong ablation of the paraffin in areas where there is no sample embedded.

The scanning rate should have been relatively high to avoid long laser incidence to NPs. Therefore, rates between 20 and 90 μm s^−1^ were investigated (LA-ICP-MS time vs intensity plots in Fig. S5, ESI), and a rate of 50 μm s^−1^ was finally selected due to good stability of signal derived from the control isotopes and the well-defined peaks derived from TiO_2_ NPs. This result is in good agreement with those previously reported which suggest the registration of discrete events minimising laser time incidence [21].

Remaining operating conditions are listed in Table 1. Regarding the dwell time, 50 ms was used for a proper acquisition of control isotopes (^25^Mg, ^26^Mg, ^27^Al, ^31^P, and ^42^Ca), whereas Ti isotopes were recorded at a dwell time of 10 ms for improving the discrimination between single-peak signals derived from TiO_2_ NP ablation and background (ionised titanium) as shown in Fig. S6.

**Table 1:** Intra- and inter-droplet variability of ^48^Ti and ^46^Ti signals from porcine gelatine standards (*n* = 5), mean slope and standard deviation of porcine gelatine-based calibrations (*n* = 3), and LOD of the method

Concentration (μg g^−1^)	^48^Ti mean intensity (cps)	RSD (%)	^46^Ti mean intensity (cps)	RSD (%)
Intra-droplet variability				
Porcine gelatine	4.87 × 10^3^	5	7.05 × 10^2^	8
0.10	1.06 × 10^4^	4	0.99 × 10^3^	12
0.20	1.59 × 10^4^	1	1.44 × 10^3^	4
0.40	2.64 × 10^4^	1	2.48 × 10^3^	3
0.80	4.62 × 10^4^	1	4.63 × 10^3^	1
2.00	9.06 × 10^4^	1	8.89 × 10^3^	1
Inter-droplet variability				
Paraffin polymer	9.70 × 10^2^	3	2.46 × 10^2^	10
Porcine gelatine	4.66 × 10^3^	6	6.67 × 10^2^	8
0.10	1.03 × 10^4^	5	1.07 × 10^3^	14
0.20	1.48 × 10^4^	1	1.39 × 10^3^	4
0.40	2.42 × 10^4^	1	2.31 × 10^3^	3
0.80	4.39 × 10^4^	2	4.33 × 10^3^	2
2.00	8.95 × 10^4^	1	8.72 × 10^3^	1
Mean slope ^48^Ti (*n* = 3)			4.21 × 10^4^ cps μg^−1^ g	
Standard deviation ^48^Ti (*n* = 3)			0.39 × 10^4^ cps μg^−1^ g	
Coefficients of determination			≥ 0.990	
LOD			0.087 μg g^−1^	

Finally, before proceeding to the imaging/mapping studies, experiments were carried out to check that 100% of the sample was ablated in a single ablation scan, and experiments based on two and three consecutive scans (two and three ablation lines over same location). Results showed that titanium records after two and three consecutive scans were negligible, and values were closed to those obtained when ablating the blanks (paraffin polymer). Therefore, we can affirm that under the optimised conditions, the embedded sample is totally ablated in one single scan.

### Analytical performances

Metals embedded in porcine gelatine standards are recommended for laser imaging of soft tissues although there are differences in the proposed procedures, mainly the heating temperature and the drop volumes used for metal embedding [20, 21, 33–35]. Before selecting the most suitable conditions to apply to the calibration strategy, the homogeneity of the drop deposited was visually evaluated when using several volumes (one drop of 2.5, 5.0, 10, and 20 μL) and when adding two successive drops. Better dispersion of the added titanium standard was found when using small drop sizes (2.5 and 5.0 μL), whereas large drop sizes led to heterogeneity and an oval-shaped dispersion, factors that increase the occurrence of bubbles in the embedded drop. Moreover, the addition of two successive drops of 2.5 μL, 5.0 μL, and 10.0 μL also led to lack of homogeneity and stability of the dispersed drop and a higher occurrence of bubbles. Best drop homogeneity was found when dispersing only one 5.0 μL drop of the titanium standards, condition that was further evaluated for establishing calibration curves and sensitivity. Homogeneity was evaluated by studying intra- and inter-droplet variabilities. Intra-day assays implied five measurements (five ablation lines) in each standard droplet (0.1, 0.2, 0.4, 0.8, and 2.0 μg g^−1^), whereas inter-day variability was assessed by ablating three times five different droplets at each titanium concentration level (0.1, 0.2, 0.4, 0.8, and 2.0 μg g^−1^). Table 1 lists the recorded ^48^Ti and ^46^Ti intensities and the relative standard deviation (RSD) of measurements, and good intra-droplet precision (RSD < 4% for ^48^Ti) and inter-droplet precision (RSD < 6% for ^48^Ti) were assessed. Therefore, the placement of 5.0-μL droplet of titanium standards in gelatine is highly homogeneous and leads to a precise calibration strategy.

**Fig. 2:**
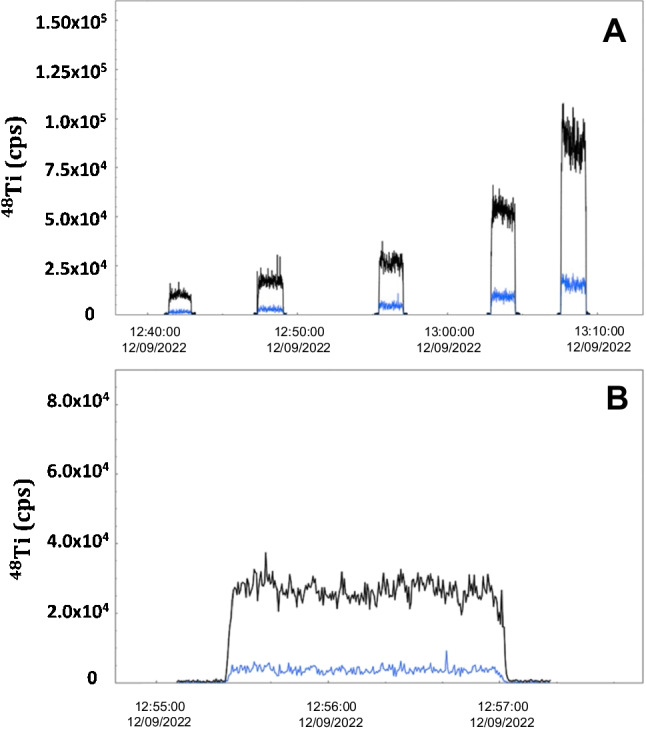
LA-ICP-MS spectra (time vs intensity) for ^48^Ti (black) and ^46^Ti (blue) in the gelatine standards displaying increasing Ti-doped concentrations: 0.1, 0.2, 0.4, 0.8, and 2.0 μg g^−1^ (from left to right in **A**). Good signal stability is observed in **B** for a standard at 0.4 μg g^−1^

Good repeatability was also observed for the prepared gelatine-based calibration since an RSD of 9% for calibration’s slope was assessed when performing three independent gelatine-based calibrations (mean slope and standard deviation of 4.21 × 10^4^ and 0.39 × 10^4^ cps μg^−1^ g, respectively). In addition, good linearity (coefficient of determination higher than 0.990) and high stability of the recorded signals (Fig. 2) were also obtained. Finally, Table 1 also lists blank values for pure gelatine which were close to those obtained when ablating the paraffin polymer used for soft tissue embedding.

Three different titanium-based gelatine calibration curves were obtained throughout the work involving ionic titanium concentrations between 0.1 and 2.0 μg g^−1^ (each standard was measured in triplicate). Table 1 lists the mean slope of the titanium-based gelatine calibration curves (4.21 × 10^4^ ± 0.39 × 10^4^), showing good repeatability [relative standard deviation (RSD) of 9 %].

The limit of detection (LOD) of the method was assessed by using the 3*σ* criteria according with

1 $$LOD=\frac{3\times Sd}{m}$$

where *Sd* is the standard deviation from eleven blank porcine gelatine measurements and *m* is the mean slope of three independent titanium-based gelatine calibration curves (4.21 × 10^4^ ± 0.39 × 10^4^ cps μg^−1^ g, Table 1) [9, 10, 36, 37]. The LOD obtained was 0.087 μg g^−1^.

### Imaging studies and spatial biodistribution of titanium

Soft tissues embedded in paraffin slides cannot be distinguished from paraffin slides under natural light, and 365-nm UV light irradiation was used to obtain clear images for distinguishing soft tissues embedded in paraffin (Fig. S2, ESI). These images were uploaded in the Iolite software, and they will be useful to correlate the sample tissue areas with the variations and recorded intensities in the LA-ICP-MS tests (verification of sample ablation when monitoring control elements such as ^26^Mg). The area of interest in the embedded samples was delimited to be close to 6 × 6 mm (length × width), which are similar to those reported in other applications [12, 14, 15, 17, 18, 36]. In order to avoid large ablation times that could drift the recorded signal, the selected areas were ablated in two separate analyses (an *y*-axis space of 75 μm between the two ablated areas), and both images were then attached in a final image.

#### Kidney tissues

**Fig. 3:**
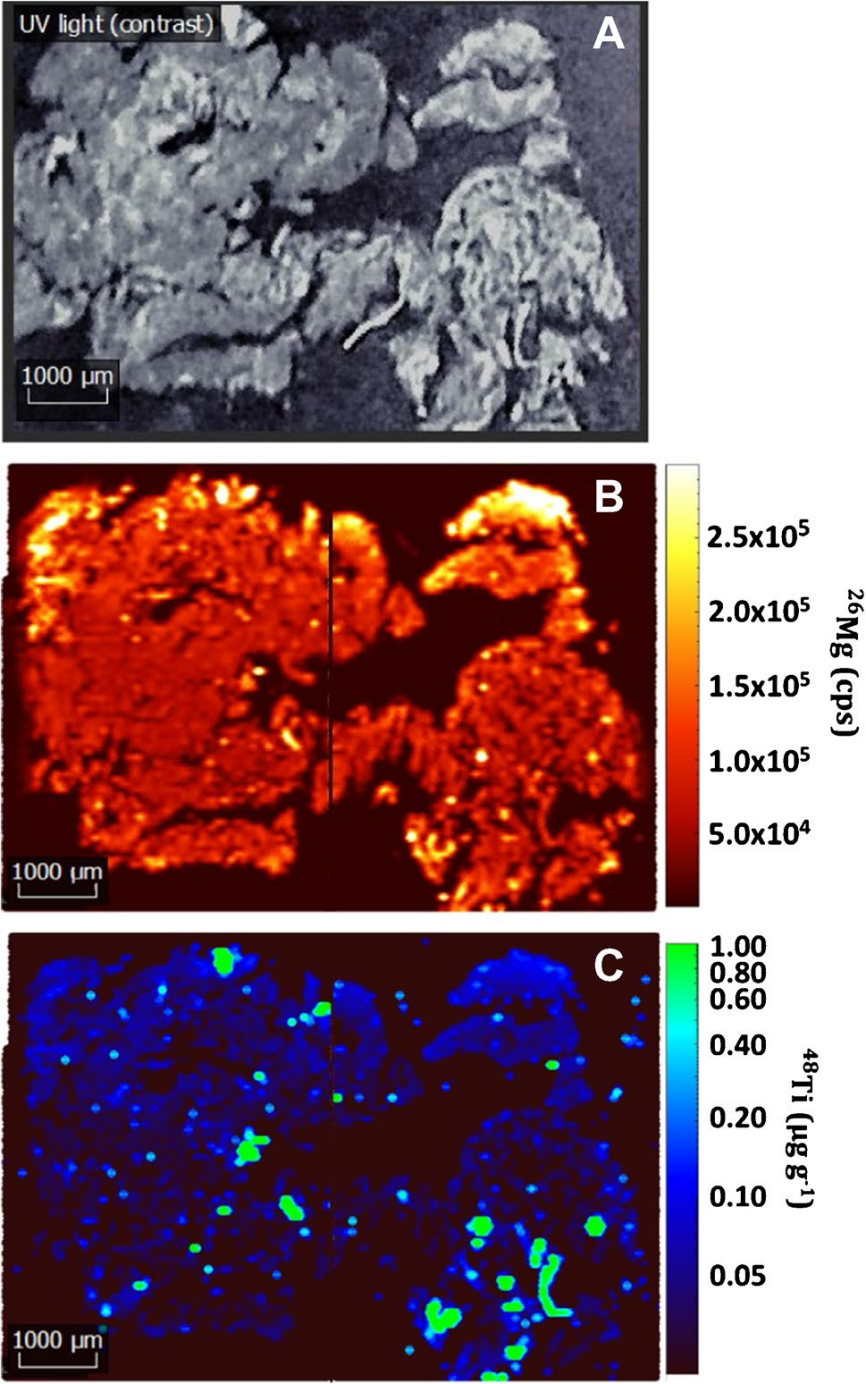
Images for a kidney tissue from a sea bream specimen exposed to 45 nm TiO_2_ NPs (dietary exposure at 1.5 mg kg^−1^) for 90-day tissues: sample image under UV light (**A**), ^26^Mg map intensities (**B**), and ^48^Ti map concentrations (**C**)

**Fig. 4:**
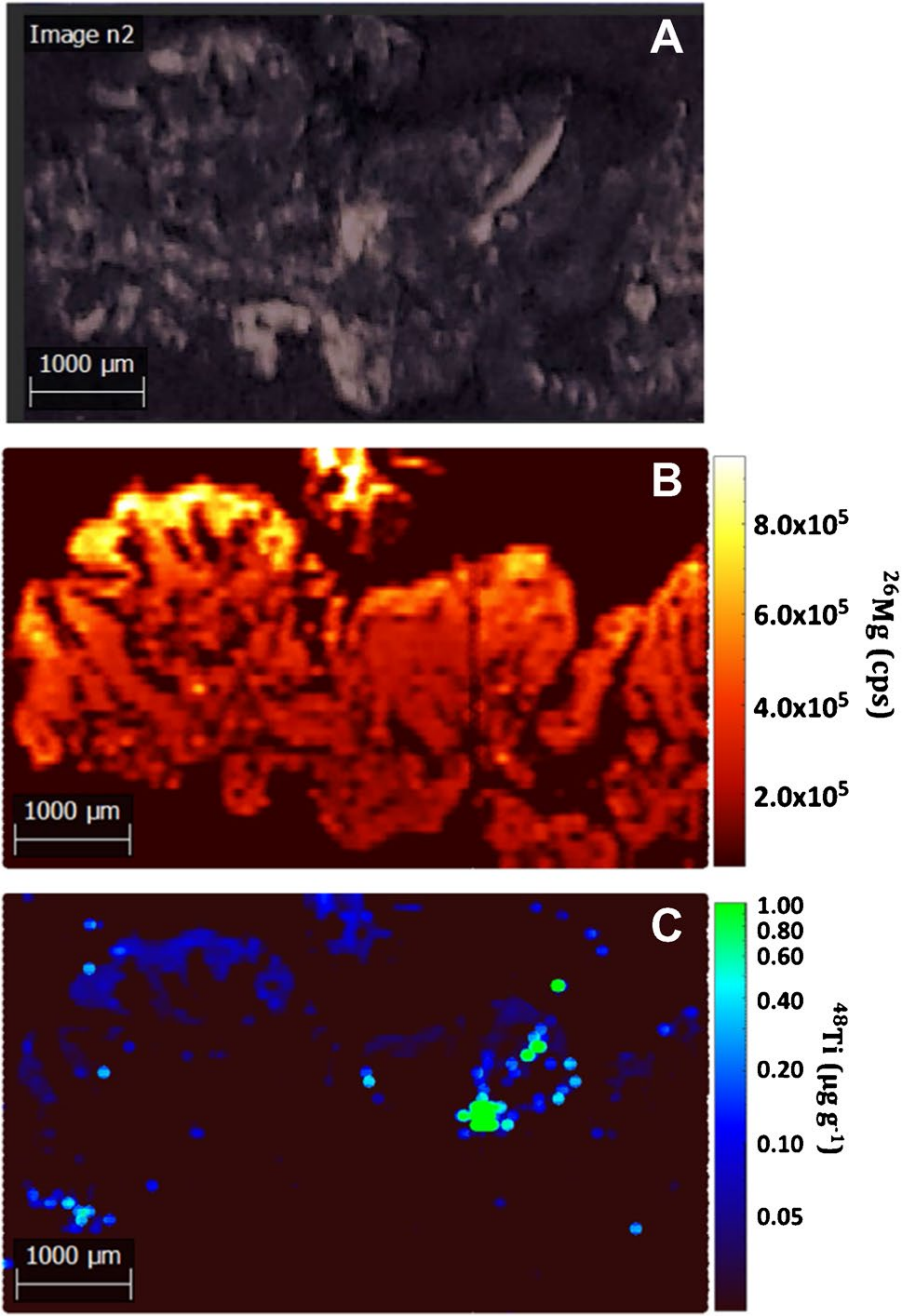
Images for a kidney tissue from an unexposed sea bream specimen (sampling at 75 days): sample image under UV light (**A**), ^26^Mg map intensities (**B**), and ^48^Ti map concentrations (**C**)

Figure 3 shows a set of 5.4 × 7.9 mm images of a kidney tissue from a sea bream previously exposed to TiO_2_ NPs, whereas Fig. 4 displays 6.0 × 3.6 mm images for a kidney tissue from an unexposed sea bream specimen (a complete set of images can be found in ESI as Figs. S7 and S8). The selected control isotope (^26^Mg in Figs. 3B and 4B) allows to distinguish the areas of the sample and to correlate this data to the UV-light images (Fig. 3A and 4A) and to the Ti distribution maps (Fig. 3C and 4C). ^26^Mg signals (Fig. 3B) were found to vary within the 1.0 × 10^5^–2.0 × 10^5^ counts per second range, whereas the images corresponding to titanium reflect some hot-spot areas, involving intense and discrete peaks which implies titanium bioaccumulation (Fig. 3C for ^48^Ti, and Fig. S7E,F for ^46^Ti). The recorded discrete signals have offered different intensities that could be attributed to both ionic titanium and TiO_2_ NP agglomerates.

Images for kidney tissues from an unexposed sea bream (Figs. 4 and S8) show clearly a lower amount of titanium throughout the tissue, and some hotspots derived from titanium bioaccumulation were only observed in a small region of the ablated sample when monitoring ^48^Ti and ^46^Ti (Figs. 4C and S8E,F). The presence of titanium in unexposed specimens may be attributed to the background titanium in sea bream and/or contamination during the exposure trials.

#### Liver tissues

Ablation areas for embedded liver tissues were 5.8 × 5.4 mm (exposed sea bream) and 8.6 × 4.6 mm (unexposed sea bream) and images are given in Figs. S9 and S10 (ESI). Different hotspots were observed in liver from TiO_2_ NP exposed sea bream (Fig. S9) although in a lower proportion than those measured in kidney tissues. The lower bioaccumulation of titanium in liver than in kidney was verified by ICP-MS after microwave acid digestion (total Ti) and by spICP-MS after enzymatic hydrolysis (TiO_2_ NPs) [38] as shown in Table S3 (ESI). Likewise, the Ti biodistribution maps, especially the hotspots, are confirmed with the use of two isotopes (Fig. S9C,E, ESI). Results for unexposed sea bream (Fig. S10, ESI) show few colour-clear spots which match with a lower titanium concentration. In addition, titanium concentration maps (Figs. S9C,E and S10C,E; ESI) are quite similar since titanium is not bioaccumulated in sea bream’s liver. These findings have been verified after assessing total titanium in the studied tissues by ICP-MS after microwave-assisted acid digestion [38] (results in Table S2, ESI).

#### Muscle tissues

Despite the difficulties for obtaining homogeneous clean cuts (slides) when using the histological sample pre-treatment for muscle tissues, the paraffin regions, and the regions embedding the sample [4.4 × 5.5 mm (exposed sea bream) and 4.4 × 5.9 mm (unexposed sea bream)] can be clearly distinguished when recording the ^26^Mg a control isotope (Figs. S11B and S12B, ESI). However, Ti-based maps for muscle tissues from exposed and unexposed sea breams (Figs. S11 and S12, ESI) have been found to be less homogeneous than those obtained for kidney and liver tissues, which is directly attributed to a less-efficient muscle embedding and microtome cuts. As expected, based on TiO_2_ NPs and total Ti determinations (Table S2, ESI), low Ti concentrations were mapped in sea bream muscle (titanium concentrations around 0.10 μg g^−1^).

## Conclusions

The optimised LA-ICP-MS methodology by using high-rate scanning and low laser energy allowed sensitive and quantitative bioimaging studies with high spatial resolution. Fast scanning has allowed to reduce signal drift problems, whereas the use of lab-produced porcine gelatine standards has been found to offer reliable capabilities for quantification and for achieving images based on concentrations rather than signals. However, although the standards’ matrix is similar to the matrix in which the tissues are embedded, there are still certain differences in the composition, and therefore differences on the ablation mechanisms are expected. Improvements are also required when applying the method to some highly brittle biological materials, such as fish muscle tissues, which leads to lack of homogeneity in the embedded samples. The use of slides with large thickness could improve the homogeneity of the embedded tissues but will require slightly higher energy to ablate the tissue in a single ablation scan and the use of longer washing times for an efficient removal of the ablated material.

The application of the developed LA-ICP-MS procedure has revealed the presence of titanium in sea bream’s tissues, and also the presence of hot spots in kidney’s tissues from exposed specimens. Therefore, quantitative element biodistribution images by LA-ICP-MS can therefore support the results obtained by other analytical techniques when assessing elements derived from inorganic nanoparticles.

## Supplementary information

ESM 1
