# Supplementary material for: Quantitative titanium imaging in fish tissues exposed to titanium dioxide nanoparticles by laser ablation-inductively coupled plasma-mass spectrometry
Source: Mikrochim Acta. 2023 Jul 18;190(8):298. doi: 10.1007/s00604-023-05895-9 (PMC10353964; doi:10.1007/s00604-023-05895-9)
Supplement: Supplementary file 1 — ESM 1 [file 604_2023_5895_MOESM1_ESM.docx]

**Electronic supplementary information**

**Quantitative titanium imaging in fish tissues exposed to titanium dioxide nanoparticles by laser ablation-inductively coupled plasma-mass spectrometry**

Cristian Suárez-Oubiña^*1^, Annarosa Mangone^2^, Lorena C. Giannossa^2^, Laura Nuñez-González^3^, Paloma Herbello-Hermelo^1^, Pilar Bermejo-Barrera^1^, Antonio Moreda-Piñeiro^1^

**Table 1**. LA-ICP-MS operating conditions.

| ICP-MS parameter (units) | Value |
| --- | --- |
| Instrument | NexIon 2000 |
| Nebulizer gas flow (L min^-1^) | 0.90 - 1.00 (daily optimised) |
| Auxiliary gas flow rate (L min^-1^) | 1.2 |
| Plasma gas flow (L min^-1^) | 15 |
| ICP RF power (W) | 1600 |
| Isotopes monitored | ^25^Mg, ^26^Mg, ^27^Al, ^31^P, ^42^Ca  ^46^Ti, ^47^Ti, ^48^Ti, ^49^Ti |
| Dwell time (ms) | 50 (^25^Mg, ^26^Mg, ^27^Al, ^31^P, ^42^Ca)  10 (^46^Ti, ^47^Ti, ^48^Ti, ^49^Ti) |
| LA-ICP-MS parameter (units) | Value |
| Laser | Nd:YAG (213 nm) |
| Tygon® interface tube | 3.5 m length – 1/16 inch i.d. |
| Ablation mode | Scan line |
| He flow rate (mL min^-1^) | 900 |
| Spot Size (µm) | 110 |
| Fluence (J cm^-2^) | 0.40 |
| Scan speed (µm s^-1^) | 50 |
| Repetition rate (Hz) | 20 |
| Ablation depth (µm) | 0 |
| Laser warm-up (s) | 10 |
| Laser wash-out (s) | 15 |

**Table S2.** Total titanium and TiO_2_ NPs in fish tissues from exposed sea bream (N1) and unexposed sea bream (N2).

| Sample tissue | sp-ICP-MS (particles g^-1^) | SD | ICP-MS (µg g^-1^) | SD |
| --- | --- | --- | --- | --- |
|  |  |  |  |  |
| Kidney N1 | 3.88×10^6^ | 1.78×10^5^ | 0.83 | 0.20 |
| Kidney N2 | --- | --- | 0.12 | 0.04 |
| Liver N1 | 6.67×10^6^ | 3.65×10^5^ | 0.51 | 0.05 |
| Liver N2 | --- | --- | 0.21 | 0.17 |
| Muscle N1 | 8.14×10^5^ | 8.90×10^4^ | 0.23 | 0.15 |
| Muscle N2 | --- | --- | < LOD | --- |

**Figure S1.** Representative TEM image of citrate-45nm TiO_2_ NPs (JEOL JEM 1010 transmission electron microscope operating at 100 kV).

**
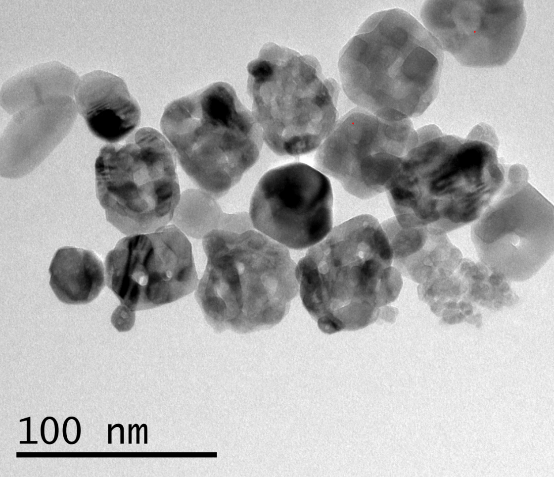
**

**Figure S2.** Thick paraffin slides (5.0 µm) of fish tissues obtained after sample pre-treatment: (A) under UV light and (B) natural light. Sample description: kidney tissue from sea bream exposed at 1.5 mg kg^-1^ TiO_2_ NPs (45 nm) for 90 days.

**Figure S3.** Exported images from ActiveView™ software (dark line under sample tissue) using laser energies for (A) sample fish tissue and (B) paraffin polymer under several laser fluencies.

**Figure S4**. Exported images from ActiveView™ software illustrating the heterogeneity of the embedded samples (A,B) and the borders between paraffin-embedded tissue / paraffin-free tissue and the glass holder (C).

**Figure S5.** LA-ICP-MS time vs intensity plots for a kidney tissue obtained at scanning rates of 30 µm s^-1^ (A), 60 µm s^-1^ (B), and 90 µm s^-1^ (C)

**Figure S6.** LA-ICP-MS time vs intensity plots for a kidney tissue by recording ^48^Ti (red), ^46^Ti (blue), and ^26^Mg (black). Scale referred only to ^46^Ti intensities: scan implying the presence of TiO_2_ NPs (A and B) and the absence of TiO_2_ NPs (C) in tissues from exposed sea bream.


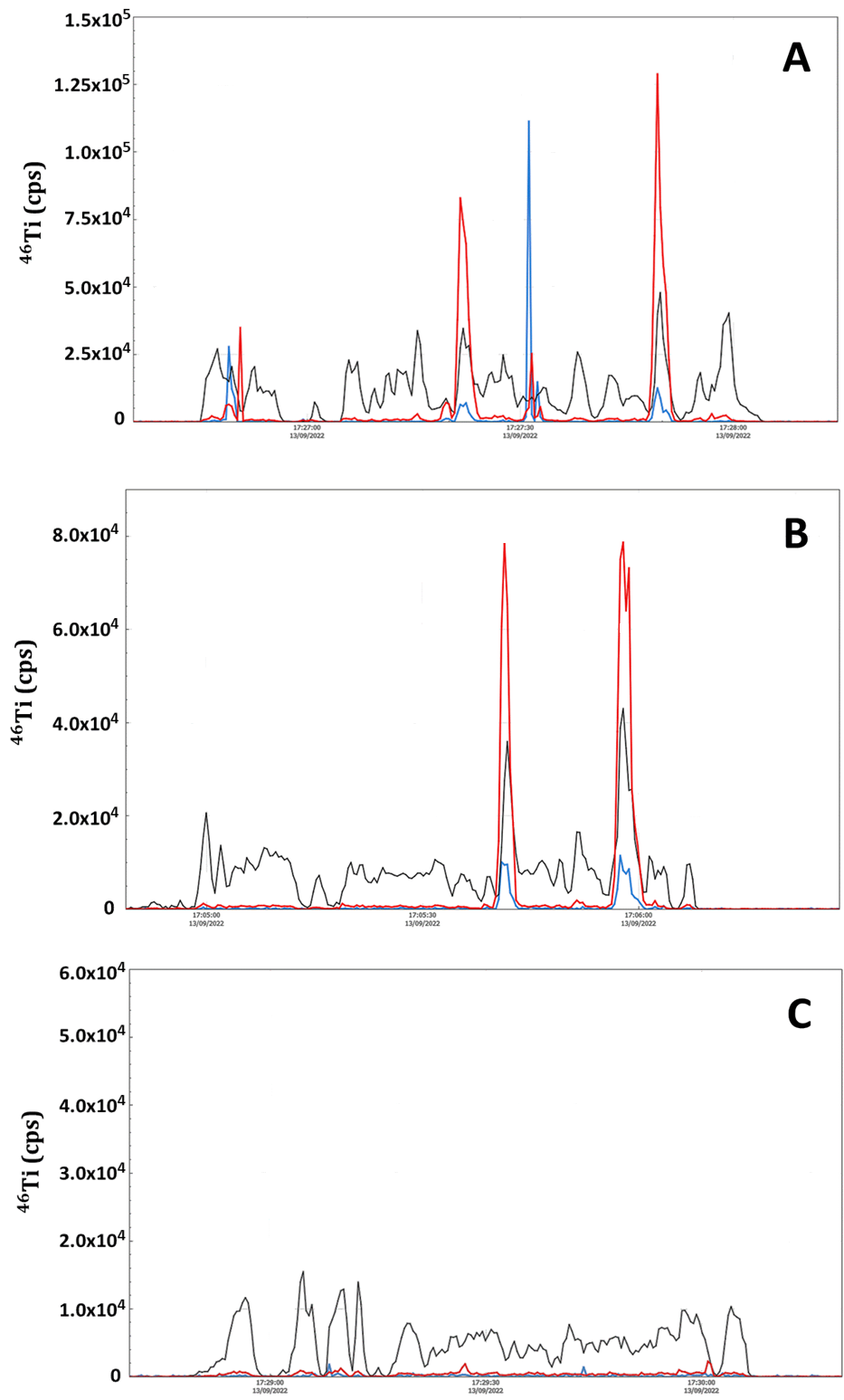


**Figure S7.** Images for a kidney tissue from a sea bream specimen exposed to 45 nm TiO_2_ NPs (dietary exposure at 1.5 mg kg^-1^) for 90 days tissues: sample image under UV light (A), ^26^Mg map intensities (B), ^48^Ti map concentrations (C), ^48^Ti map intensities (D), ^46^Ti map concentrations (E), ^46^Ti map intensities (F).

**Figure S8.** Images for a kidney tissue from an un-exposed sea bream specimen (sampling at 75 days): sample image under UV light (A), ^26^Mg map intensities (B), ^48^Ti map concentrations (C), ^48^Ti map intensities (D), ^46^Ti map concentrations (E), ^46^Ti map intensities (F).

**Figure S9.** Images for a liver tissue from a sea bream specimen exposed to 45 nm TiO_2_ NPs (dietary exposure at 1.5 mg kg^-1^) for 75 days tissues: sample image under UV light (A), ^26^Mg map intensities (B), ^48^Ti map concentrations (C), ^48^Ti map intensities (D), ^46^Ti map concentrations (E), ^46^Ti map intensities (F).

**Figure S10.** Images for a liver tissue from an un-exposed sea bream specimen (sampling at 45 days): sample image under UV light (A), ^26^Mg map intensities (B), ^48^Ti map concentrations (C), ^48^Ti map intensities (D), ^46^Ti map concentrations (E), ^46^Ti map intensities (F).

**Figure S11.** Images for a muscle tissue from a sea bream specimen exposed to 45 nm TiO_2_ NPs (dietary exposure at 1.5 mg kg^-1^) for 75 days tissues: sample image under UV light (A), ^26^Mg map intensities (B), ^48^Ti map concentrations (C), ^48^Ti map intensities (D), ^46^Ti map concentrations (E), ^46^Ti map intensities (F).

**Figure S12.** Images for a liver tissue from an un-exposed sea bream specimen (sampling at 45 days): sample image under UV light (A), ^26^Mg map intensities (B), ^48^Ti map concentrations (C), ^48^Ti map intensities (D), ^46^Ti map concentrations (E), ^46^Ti map intensities (F).
